# Collagen crosslinking-induced corneal morphological changes: a three-dimensional light sheet Microscopy-based evaluation

**DOI:** 10.1038/s41598-024-78516-x

**Published:** 2024-11-16

**Authors:** Axel Stoecker, Diana Pinkert-Leetsch, Timea Koch, Roland Ackermann, Stefan Nolte, Christian van Oterendorp, Christoph Russmann, Jeannine Missbach-Guentner

**Affiliations:** 1 Faculty of Engineering and Health, University of Applied Science and Arts, 37085 Goettingen, Germany; 2 Department of Diagnostic and Interventional Radiology, University Medical Center Göttingen, Goettingen, 37075 Germany; 3 Institute of Applied Physics, Abbe Center of Photonics, Friedrich-Schiller-Universität Jena, Jena, 07745 Germany; 4 Fraunhofer Institute for Applied Optics and Precision Engineering IOF Jena, Jena, 07745 Germany; 5 Department of Ophthalmology, University Medical Center Göttingen, Goettingen, 37075 Germany; 6 Molecular-Biomarkers-Nanoimaging Laboratory (MBNI), Brigham & Women’s Hospital Harvard Medical School, Boston, MA USA

**Keywords:** Extracellular matrix stiffness, Corneal stroma, Light sheet microscopy, Tomographic microscopy, Autofluorescence, 3D virtual histology, Diseases, Medical research

## Abstract

Stiffness-related eye diseases such as keratoconus require comprehensive visualization of the complex morphological matrix changes. The aim of this study was to use three-dimensional (3D) light sheet fluorescence microscopy (LSFM) to analyze unlabeled corneal tissue samples, qualitatively visualizing changes in corneal stiffness. Isolated porcine corneal tissue samples were treated with either NaCl or 0.1% glutaraldehyde (GTA) prior to clearing with benzyl alcohol/benzyl benzoate (BABB) and subsequently scanned with LSFM. After analysis of the LSFM data sets, the samples were embedded in paraffin to validate the results by conventional planar microscopy. In the unlabeled corneal tissue samples the 2D/3D morphology of the entire tissue volume was identified by specific autofluorescence signals. An enhancement of collagen crosslinking was induced by applying GTA to the corneal tissue. Subsequent LSFM scans showed specific morphological changes due to altered autofluorescence signals of the corneal stroma, which were confirmed by conventional histology. Therefore, LSFM analysis of corneal tissue samples allowed label-free 3D autofluorescence assessment of the corneal morphology in its anatomical context. It provides the technical basis for the examination of the pathologically altered cornea and facilitates ophthalmologic examinations of corneal diseases based on the altered tissue stiffness.

## Introduction

The cornea is responsible for about 2/3 of the refractive power of the eye^1^. The unique composition of the corneal stroma of mainly type-1 collagen fibers^2^provides transparency and stability in equal measure. If the corneal connective tissue is too thin, keratoconus (KC) may develop as an ectatic disorder^3^. Progression of the disease leads to increasing visual impairment and is one of the three most common indications for corneal transplantation worldwide^4^.

For about 20 years, UV-A-riboflavin crosslinking^3,5,6^has been used to stabilize weak corneal tissue in KC and corneal incisions^7,8^after refractive surgery. The riboflavin triplets generated by UV-A radiation are capable of forming active oxygen and riboflavin radicals, which react with the side chains of the amino acids of the collagen molecules, forming covalent bonds between them. As a result, the diameter of the collagen fibers become larger and the mechanical stiffness increases^5,6^.

Research on KC and other ectatic corneal diseases addresses not only biomechanical investigations of corneal elasticity but also aspects of aging, hereditary predisposition, secondary diseases and strategies to improve and further develop established techniques of refractive surgery. There is a need for suitable models to visualize the cornea and its alterations and evaluate novel therapeutic strategies. In contrast, there are few suitable animal models to represent the progression of rigidity associated corneal diseases and to evaluate innovative interventions. In the absence of an appropriate disease model, manipulation of corneal stiffness, such as corneal application of collagenase-2 in a rabbit model, is required^8,9^. However, in addition to compatible disease models, visualization of altered corneal rigidity is also needed for an accurate evaluation of innovative therapeutic interventions.

One option for comprehensive three-dimensional (3D) imaging is fluorescence-based light sheet microscopy (LSFM), which provides a 3D tissue topography based on laser excitation. To visualize an entire volume, laser light sheets are used for 2D fluorescence imaging, generating tomographic layer-by-layer data sets of the sample volume^10^. The penetration depth is mainly limited due to scattering in the tissue. To increase the penetration depth of the light sheet, tissue transparency must first be achieved during a tissue clearing step, including the adjustment of the refractive index^11–13^. LSFM has shown its outstanding value in the study of tissue specimens, especially in terms of their interaction with the environment in 3D with or without fluorescent labeling^13–16^. The technique of tissue clearing and 3D visualization of murine eyes using LSFM has already been successfully established^17,18^. On murine intact eyeballs, neovascularization after inflammation could thus be visualized^19^as well as retinal vascular development on rat eyes using LSFM^20^.

The purpose of this study was to address the question of whether LSFM is also capable of imaging ultrastructural changes in the cornea and to clarify whether LSFM is a suitable tool for analyzing preclinical ophthalmic models and rigidity-related diseases. Therefore, the histological morphology of corneal tissue in a label-free and spatial manner was depicted using LSFM. By this ex vivo examination method, we visualize structural changes of the corneal stroma as changes in the autofluorescence of collagen induced by glutaraldehyde (GTA) in all three dimensions of the tissue. For this purpose, the effect of GTA was investigated in the corneal tissue^21^. Although GTA as a bifunctional crosslinking agent differs in the mechanism of protein crosslinking compared to riboflavin UV-A crosslinking^22,23^, the results of both types of crosslink exhibit a comparable stiffness of the cornea^21^. In general, it is assumed that protein crosslinking using GTA leads to the formation of Schiff bases^24^.

The results of the study show that 3D LSFM can accurately represent qualitative changes in stromal organization as a prerequisite for the evaluation of novel treatments for rigidity associated corneal diseases.

## Results

### LSFM corneal data reveals extensive fluorescence changes after GTA treatment

The main aim of this study was to investigate whether changes in cross-linking-induced tissue morphology can be identified and visualized by LSFM. For this purpose, corneal biopsy samples were treated ex vivo with 0.1% GTA and compared with untreated corneal samples.

Previous studies have demonstrated that the use of GTA led to a crosslinking of stromal collagen fibrils with a comparable result to corneal crosslinking by riboflavin and UV light in clinical ophthalmology^21^.

**Fig. 1:**
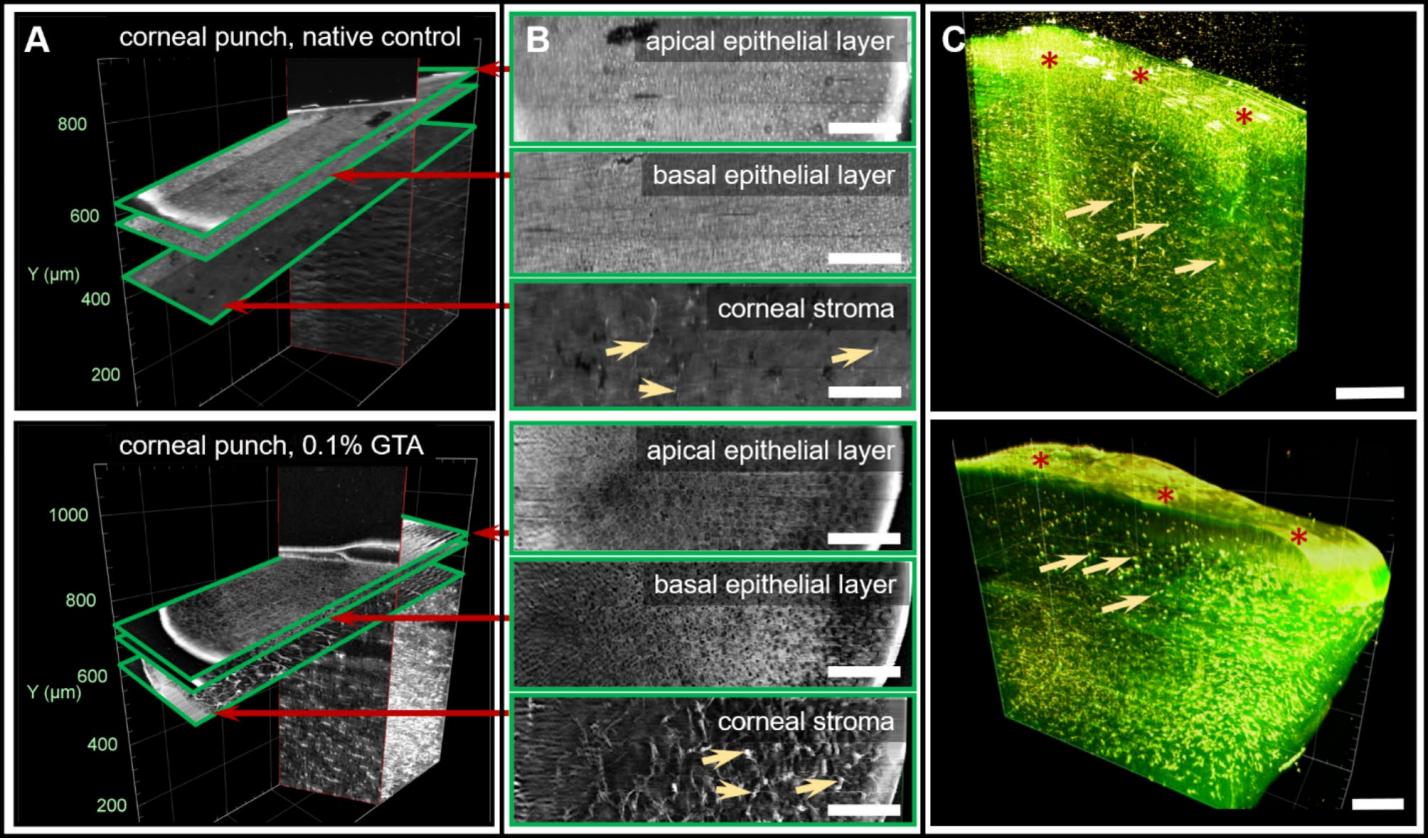
Virtual sections of LSFM datasets of corneal tissue samples with intact epithelium (red asterisk). From 3D rendering of the corneal images (**A**), 2D planes of the volume were generated to analyze comparable tissue sections. As shown in rendered 2D (**B**) and colored 3D images (**C**) the expression of keratocytes (arrows) in emission with center wavelength λ_em_ = 585 nm (yellow) and the surrounding collagen matrix with emission at λ_em_ = 525 nm (green) changes with GTA treatment. The extracellular matrix and nuclei of keratocytes (arrows) showed an enhanced, but heterogeneously distributed fluorescence in comparison to the homogeneous fluorescent corneal stroma of NaCl-treated corneal tissue samples. The high-resolution cutout images were taken from corneal tissue samples with epithelium, native and 0.1% GTA treated. Scale bar = 200 μm.

In the NaCl-treated control group, corneal tissue samples revealed the highly fluorescent epithelium of the cornea in the corresponding 2D and 3D data sets with both filter combinations (Fig. 1). The directly adjacent stromal portion of the cornea emitted short wavelength light in the green range as a uniformly distributed signal corresponding to the collagenous component and therefore was clearly distinguishable from the surrounding structures. In addition to this basic fluorescence, there were also abundant distinct fluorescent spots corresponding in morphology to the nuclei of keratocytes (Fig. 1A). The uniform distribution of keratocyte nuclei within the corneal stroma was observed over the entire 3D volume of the tissue sample.

The 0.1% GTA-treatment showed a clear increase of autofluorescence in the periphery of the sample volume (Fig. 1B) signing a gradual penetration of the corneal tissue sample with GTA due to an incomplete diffusion within time of 10 min of treatment.

Because of this strong autofluorescence, even the fluorescence of the keratocyte nuclei within the peripheral stroma was difficult to determine (Fig. 1B). It was not the amount of keratocytes that changed, but their distribution in the direction of the sample periphery. To assess this effect more accurately, virtual 2D tomographic slices were obtained in parallel to the stratification of the cornea and compared with one another (Fig. 1). While the NaCl control and the 0.1% GTA tissue samples showed moderate fluorescence in the epithelial region, the underlying stromal layer displayed an increased fluorescence after treatment with 0.1% GTA in the LSFM data set in comparison to control samples (Fig. 1B). In particular, the distribution of keratocyte nuclei and the autofluorescent collagen fibers within the corneal stroma showed the same gradual decrease of fluorescence from the punch periphery toward the inner portion after GTA treatment as revealed in 3D. In contrast, in the NaCl-treated control tomographic images, a homogeneous distribution pattern of the keratocyte nuclei and collagen fibers were observed (Fig. 1). Thus, in both 3D and 2D LSFM data sets of NaCl control and 0.1% GTA treated tissue samples morphological differences were based on a precise spatial distribution of cells and collagenous matrix and their altered autofluorescence properties.

### Plate reader analysis reveals GTA-enhanced autofluorescence of collagen

To quantify the autofluorescence of NaCl and GTA treated corneal tissue, the treated samples were evaluated in a plate reader prior to LSFM processing. The aim of this alternative fluorescence measurement was to validate whether the increase in autofluorescence in the 0.1% GTA treated tissue samples observed via LSFM was detectable by approved methods.

**Fig. 2:**
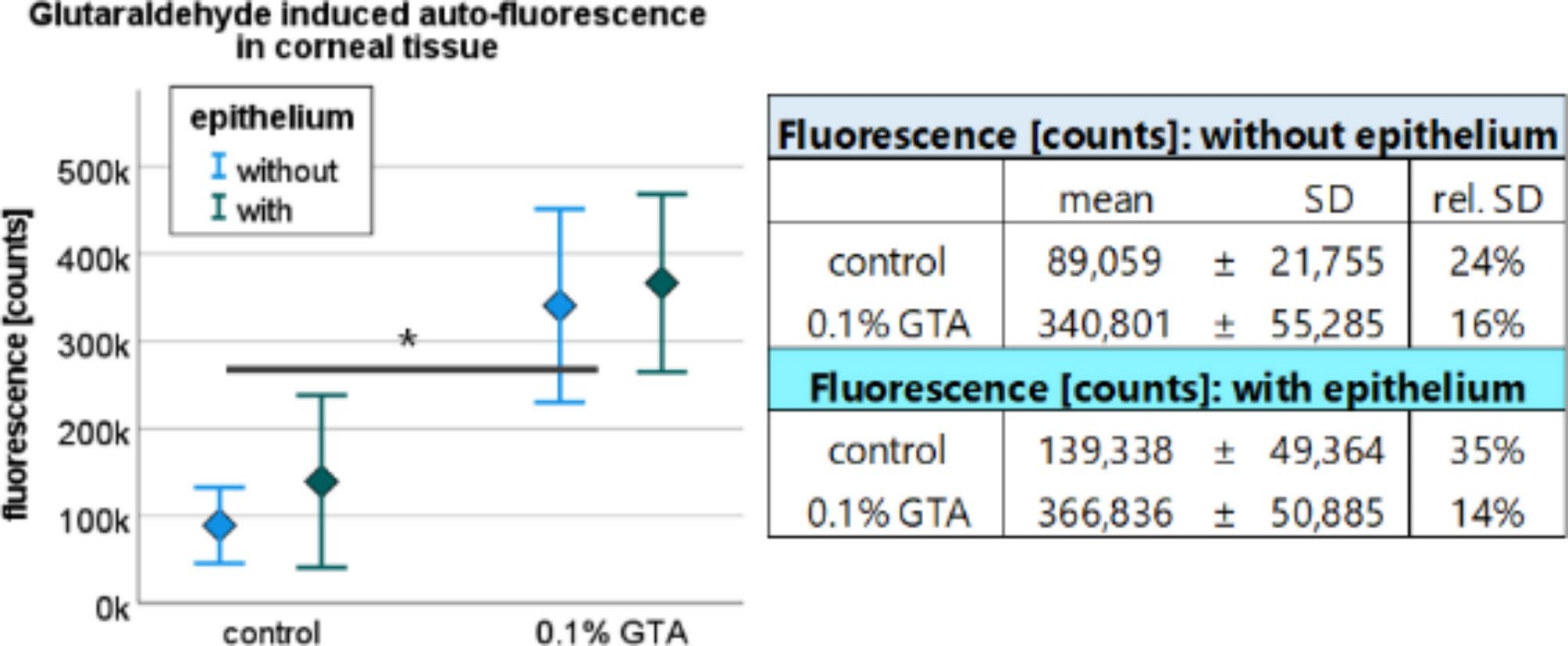
Quantification of autofluorescence in GTA-treated corneal tissue. Samples (*n* = 12) either without or with epithelium were treated with NaCl as control or 0.1% GTA. Regardless of epithelial presence, fluorescence increased when using GTA (* *p* < 0.02). Dots and whiskers represent mean value and standard deviation (SD).

For GTA treated samples without epithelium, autofluorescence intensity was measured with I_GTA_ = (340,801 ± 55,285) counts (Fig. 2). The GTA induced fluorescent intensity differed from the measured fluorescence intensity of untreated samples I_control_ = (89,059 ± 21,755) counts with a significance of *p* < 0.02. As shown in Fig. 2, the fluorescence signal of the tissue samples with epithelium was measured in the range of the standard deviation of the tissue samples without epithelium. It was considered equal with a probability of p_GTA_ > 0.5. These results confirmed the increase in fluorescence intensity in 0.1% GTA-treated corneal tissue samples compared to control samples as observed in the LSFM data sets.

### The characteristic morphology of the cornea, revealed by autofluorescence is comparable to conventional histology

**Fig. 3:**
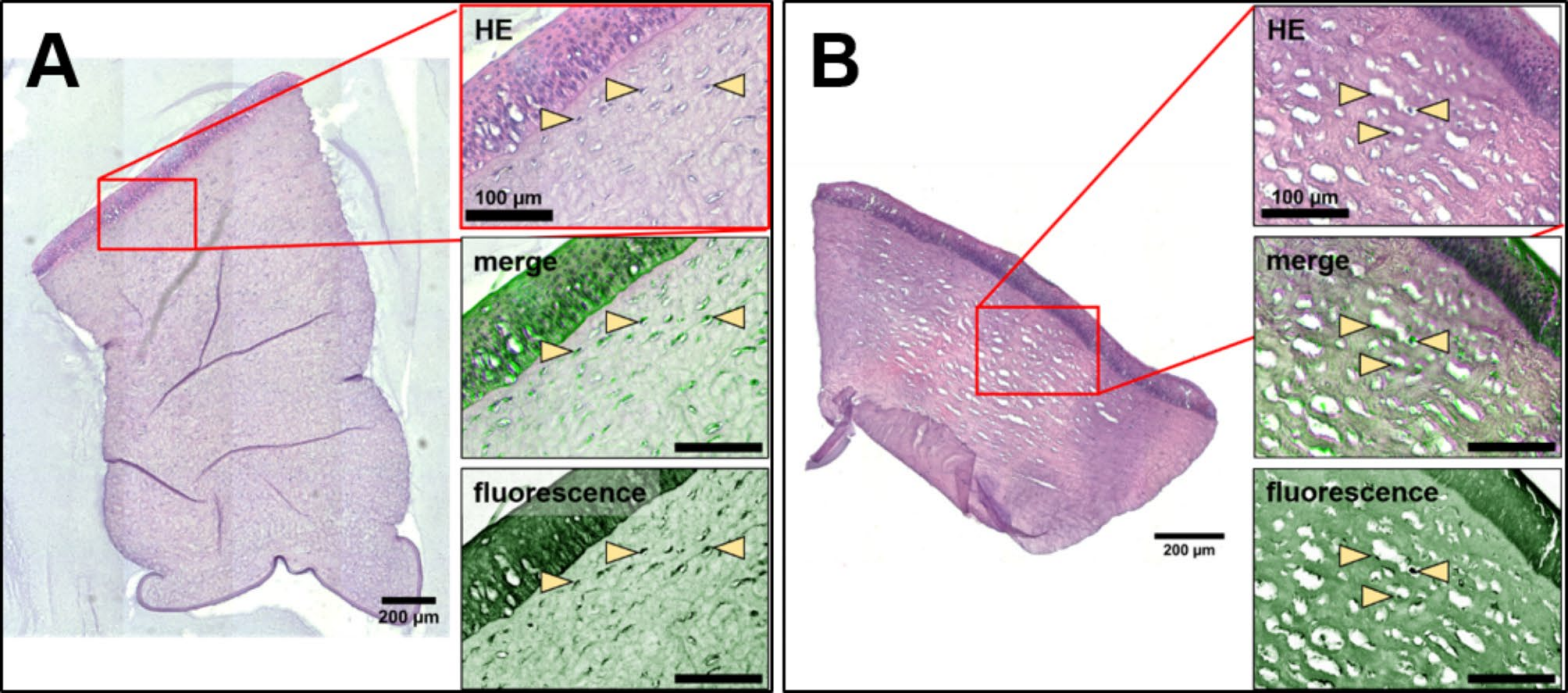
Alignment of planar H&E sections with their corresponding fluorescent microscopic images (inverted, green) in a native corneal tissue punch (**A**) and a 0,1% GTA treated corneal tissue punch (**B**). The H&E staining revealed the morphology of the multi-layered epithelium and the corneal stroma with scattered keratocytes (arrows). These structures could be clearly linked to the signals within the fluorescence image (arrows). Due to the inverted visualization, enhanced fluorescence of collagen fibers and nuclei in the GTA treated sample appear as a dark green stain and dark spots in the fluorescence image. A merge of the confocal microscopy and the H&E images confirmed a clear localization of the fluorescence within the nuclei of epithelial cells and keratocytes.

To confirm the morphological structures of the cornea identified by LSFM, the corneal tissue samples were paraffinized and histological sections of the cleared corneal samples were prepared. Adjacent slices were independently processed (i) for planar light microscopy using chemical stained sections and (ii) for 2D fluorescence microscopy using native, unlabeled sections (Fig. 3).

Since the fluorescence microscopic sections correspond to a high-resolution 2D representation of the LSFM, the fluorescence signals detected here served as a comparison pattern for the LSFM data. Control corneal tissue samples as well as samples treated with 0.1% GTA were compared with each other (Fig. 3).

**Fig. 4:**
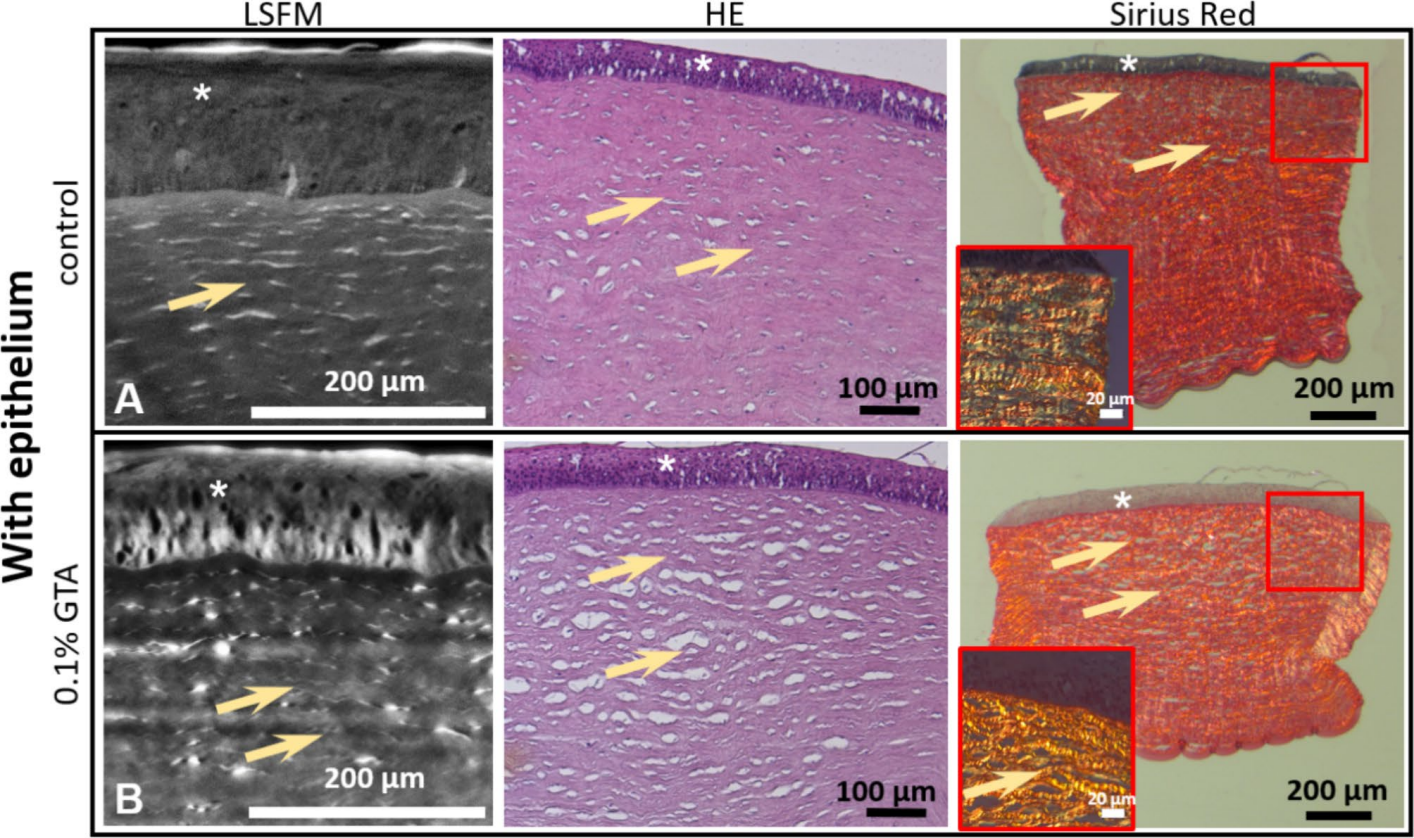
Comparison of planar images of NaCl control and GTA-treated corneal tissue samples generated by LSFM or adjacent tissue sections stained with H&E or Sirius red. **(A**) The LSFM autofluorescence image of the NaCl-treated control corneal punch as a tomographic image section of the upper corneal area including the epithelium (asterisk) shows a clear but weak fluorescence in the epithelium and a homogeneous fluorescence within the collagen matrix of the stroma caused by the evenly distributed collagen fibers (arrows) and some keratocyte nuclei. Subsequent staining of individual sections with H&E and Sirius red shows the uniform distribution of the corneal stroma with individual keratocytes and horizontally and parallel aligned collagen fibers (Sirius Red, insert). (**B**) After treatment with 0.1% GTA, the fluorescence of the epithelium (asterisks) is increased in the nuclei. The corneal stroma shows a clear increase in fluorescence, which originates in particular from keratocyte nuclei and collagen fibers. The subsequent staining of individual sections with H&E and Sirius red shows an altered morphology of the collagen fibers in the honeycomb shape, which are more condensed than in the control and thus have a stronger polarization (Sirius red, insert).

A clear stratification of the cornea with the apically located multilayered epithelium was identified in both: H&E staining and fluorescence microscopy, based on the autofluorescence of the nuclei, basement membrane and the collagenous stroma. The autofluorescence properties of the nuclei also resulted in an unequivocal identification of keratocytes within the corneal stroma. A merge of the H&E stained section and the fluorescence image confirmed the distinct autofluorescence signal in the corneal stroma as keratocyte nuclei (Fig. 3). The extracellular matrix around the keratocytes also showed homogeneous autofluorescence signals corresponding to the amount and distribution of collagens (Fig. 4).

In the GTA-treated tissue samples (Fig. 3B), a stronger autofluorescence based on the enhanced fluorescence of the keratocyte nuclei and the collagen fibers of the corneal stroma was observed than in the NaCl-treated sample (Fig. 3A). These results were consistent with the analyzed density and distribution of keratocytes in the LSFM data sets (Fig. 1).

### 2D LSFM representation of the cornea identifies complementary information of morphological alterations in comparison to conventional histological staining

As shown in the previous visualizations of LSFM images of the cornea, the collagen matrix within the corneal stroma was clearly visible and emitted autofluorescence signals at λ_em_ = 525 nm, as did the apical epithelium (Fig. 1). Structural differences due to chemically induced crosslinking of stromal collagen led to an enhancement of autofluorescence from collagen fibers and keratocyte nuclei (Figs. 1 and 3).

To determine whether the LSFM data can be validated by 2D light microscopy and provide additional information to conventional planar light microscopy, sections of the cleared corneal tissue samples were prepared and stained sequentially with H&E and Sirius red. In the virtual section of the LSFM dataset of NaCl-treated corneal tissue, the stroma showed a uniformly parallel collagen fiber arrangement and homogeneously distributed keratocyte nuclei. When stained with Sirius red using a polarizing filter, the individual bright collagen fibers within the corneal stroma and Descemet’s membrane appeared as a homogeneous, parallel-fibered layer (Fig. 4A).

High-resolution LSFM images of tissue samples treated with GTA showed strong fluorescence in the nuclei of epithelial cells and keratocytes. The collagen fibers of the stroma appeared more fluorescent, but more heterogeneously than in the NaCl-treated sample. (Fig. 4B). In H&E staining, the GTA-treated corneal tissue samples showed wide cavities within the collagen-containing stroma (Fig. 4B). Although the epithelium and Descemet’s membrane appeared unremarkable compared to the NaCl controls, in Sirius red staining with a polarizing filter revealed a brighter appearance of stromal collagen of GTA treated samples. The cavities already observed in the H&E staining were also visible. This “honeycomb shape” collagen signature is an expression of stronger collagen crosslinking and is associated with increased tissue stiffness^25^. Sirius red staining in combination with polarized light microscopy is strictly selective for collagen due to the birefringent properties of collagen fibers^26^. Therefore, the GTA treated sample is affirming stronger collagen interconnections^26,27^ due to the color shift from the prominent green color in the NaCl treated tissue sample towards orange after GTA treatment.

## Discussion

This study aimed to visualize the histological morphology of corneal tissue and its alterations label-free and spatially using LSFM. With this ex vivo examination method, we visualize changes in autofluorescence patterns in the corneal stroma due to chemically induced collagen modifications in all three dimensions. The application of GTA as an inducer of collagen crosslinks was investigated for qualitative changes in stromal organization^21^. The results of this study show that 3D LSFM is a suitable tool for the analysis of distinct morphological alterations of the corneal stroma. This is of particular value in the evaluation of preclinical ophthalmic models, for example in studies of keratoconus or wound healing.

We have demonstrated a virtual histology approach on isolated corneal tissue samples to obtain a 3D dataset with a detailed representation of the structural and morphological features of the cornea. Compared to the planar histological single section technique, 3D LSFM datasets allow the visualization of the entire tissue volume in any plane and angle.

Approaches of 3D fluorescence-based tomographic microscopy have already been realized to visualize murine organs such as testes without extrinsic fluorophores^16^or to enable the identification of tumor margins^14,15^. LSFM has also found its way into ophthalmologic research. Singh and colleagues used the marker-free 3D microscopic approach to observe the development of retinal vessels in a rodent model^20^, while immunofluorescence labeling was used to visualize choroidal and retinal vessel density^18^and the microvasculature of the iris and posterior bulb^28^ in mice.

To the best of our knowledge, no three-dimensional (3D) light sheet fluorescence microscopy (LSFM) study has been conducted on the visualization of collagen crosslinking in the corneal stroma.

Label-free LSFM in combination with the use of different filter combinations offered the possibility to discriminate ultrastructural changes due to altered collagen crosslinks of the porcine cornea. These morphological changes were detected based on a precise spatial distribution of cells and collagenous matrix and their altered autofluorescence properties.

We showed that chemical treatment of corneal tissue samples with GTA leads to a structural change and therefore an alteration of the autofluorescence properties within the corneal stroma, which was confirmed by plate reader examination.

Spoerl and colleagues showed in their research for the Dresden protocol, that the treatment of corneal tissue with GTA leads to a higher tissue stiffness^21^. Following their treatment protocol we can assume, that the increase of autofluorescence could be directly connected to a higher tissue rigidity. Therefore, we showed that chemical treatment of corneal tissue samples with GTA leads to a structural change and a corresponding alteration of the autofluorescence properties within the corneal stroma.

Although the alteration in autofluorescence properties of corneal collagen following GTA treatment could be qualitatively assessed using LSFM, a quantitative evaluation, such as the determination of a specific change in the autofluorescence spectrum of collagen before and after crosslinking, was not feasible using LSFM and is currently the subject of ongoing research. The analysis of the spectral fluorescence properties after corneal collagen crosslinking would provide an opportunity to gain a better understanding of the molecular effects of crosslinking and to investigate dose effects more thoroughly. The validating use of a plate reader confirmed the alteration in autofluorescence following collagen crosslinking.

Although the plate reader data did not reveal the spatial distribution of autofluorescence within the corneal tissue samples, they provided a quantitative value of the overall fluorescence characteristics. Treatment with 0.1% GTA showed a fourfold increase in total fluorescence compared to the control group, regardless of the presence of the epithelium.

Additionally, the histological sections revealed an altered collagen fiber arrangement after GTA treatment. Since it has been shown for various tumor lesions that altered collagen fiber arrangements correlate with tissue stiffness, it can also be concluded that the honeycomb collagen formation observed in the GTA samples is associated with stiffer corneal tissue^25,29^.

Bohn and colleagues made a similar observation in the in vivo histology approach of the cornea using the *Rostock Electronic slit lamp*^30^. For this purpose, a confocal in vivo microscope was used, which is placed on the cornea and to tomographically scan the entire cornea. In contrast to healthy volunteers, patients after corneal crosslink with the Dresden protocol showed differences in the morphology of corneal stroma. The fluorescence of keratocyte nuclei and cytoplasm was increased after corneal crosslink. This cellular appearance was described by the authors as hyperreflective and can be understood as a reaction to the crosslink, in accordance with our findings.

Although the collagen matrix exhibits more homogeneous fluorescence at lower excitation wavelengths, visual inspection of the LSFM data indicates that keratocytes are more distinguishable at higher excitation wavelengths.

However, the differences in excitation wavelengths are too subtle to enable reliable discrimination between the two distinct sources of autofluorescence. A reliable means of distinguishing between collagen fibers and keratocyte nuclei would be to determine the spectral fingerprint of both morphological structures, which is not feasible with the LSFM used.

LSFM data provides 3D and 2D tomographic information on structural and morphological features based on their autofluorescence properties. This is a distinct advantage over histological staining techniques such as H&E or Sirius red. Especially the Sirius red staining method is highly sensitive to even minor changes regarding the colour distribution when examined under polarised light. This high interobserver variability demands therefore a strict standardization of microscopic parameters to obtain reliable results^31^.

As demonstrated previously, performing tissue clearing and LSFM imaging does not prevent subsequent histological staining, which provides planar information on the cellular morphology in both healthy and altered tissue. In addition, a previous study on whole murine testes confirmed that immunohistochemical (IHC) staining of the CD31 antibody after tissue clearing with BABB produced the same staining patterns as in untreated, paraffin-embedded tissue^16^. This allows both analytical approaches: LSFM and histology or IHC to be performed on the same sample. One does not preclude the other.

An alternative, frequently utilized imaging modality for visualizing the eye at subcellular resolution is two-photon microscopy (2P-microscopy), which avails the tissue inherent autofluorescence potential of tissue structures. In addition to images of autofluorescence, it also offers the possibility of second harmonic generation (SHG) to image non-centrosymmetric structures such as fibrillar collagen type-I and collagen type-II^32^. By using this method as an ex vivo approach, the gain of high-resolution information for structure recognition in the eye is enormous.

In addition to histological identification of the peripheral corneal region^33^and precise differentiation between collagen and elastin with 2P excitation fluorescence microscopy, 2P-microscopy was able to visualize diseased human corneas and showed distinct SHG behavior for corneas affected by keratoconus and keratitis^34,35^. In a further step, after riboflavin crosslink in corneal tissue samples, the authors were able to assess not only the change in autofluorescence but also the fluorescence lifetime as a parameter of crosslink efficacy^36^. Without doubt, ex vivo 2P-microscopy is superior to LSFM in terms of spatial resolution and less light scatter^28^. However, this also means a smaller image section, a longer time for image acquisition and a larger data set in comparison to equivalent LSFM specimens. The specific autofluorescence or second harmonic generation signals can also be detected in vivo using 2P-microscopy in an eyeball microscope contact method^37^. Overall, the images are two-dimensional and a subcellular resolution of the epithelium and the corneal stroma is not achieved in this study.

We demonstrated that LSFM can facilitate virtual sectioning with the assistance of complete volume datasets. It can provide a new understanding of cellular organization in whole organs and opens opportunities to trace sub-structures and their fluorescence properties over the entire volume. As a result, LSFM can help to support digital image registration and artificial intelligence-based image processing, for example.

In the future, ophthalmologic studies of corneal aging, stiffening or thinning will benefit from the ability to measure corneal structural changes using 3D data and modified autofluorescence. Newly emerging techniques for visualizing and measuring rheological and biomechanical characteristics of the cornea, such as spontaneous and stimulated Brillouin spectroscopy^38^, will expand our understanding and also the diagnosis of corneal diseases. This will help in the development of new, required treatment approaches. The technical and instrumental prerequisites for this research are now in place.

## Materials and methods

### Tissue sample acquisition and processing

For this study, we have received already euthanized minipigs (Goettingen minipigs, age 2–4 years) from third parties. They were euthanized through deep anaesthesia with pentobarbital (250 mg/kg bodyweight). For the ex vivo examinations to be performed, the eyes were dissected, transferred to sodium chloride solution (NaCl, 0.9%), and stored on ice for a maximum of 2 h until further use.

Out of eight eyes, the uppermost corneal layer (epithelium) was removed from every second eyeball using a hockey knife. Subsequent preparation of the cornea and all further experimental work was performed at room temperature. Unless otherwise noted, processed specimens were placed in NaCl solution to prevent them from drying.

To obtain comparable results, six tissue samples per cornea were collected using a 2 mm biopsy punch and incubated in triplicate in black 96-well plates (flat bottom). Referring to Spoerl et al. the tissue samples were treated with either NaCl or 0.1% glutaraldehyde (GTA) in phosphate buffered saline (PBS) solution for 10 min^21^. The anterior surface of the cornea pointed upward, so the fluids could only penetrate from top and the sides.

This resulted in four groups of 12 tissue samples: NaCL treated and without epithelium, GTA treated and without epithelium, NaCL treated with epithelium and GTA treated with epithelium. Overall, we analyzed 48 tissue samples. Tissue samples were taken randomly from the individual cornea (Table 1).

**Table 1:**
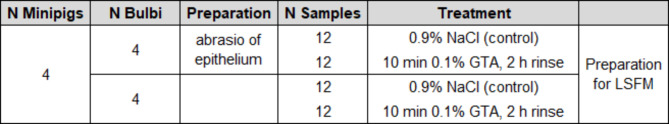
Detailed overview of numbers of eyes, tissue samples and treatment combinations.

After rinsing the samples two times for one hour with NaCl solution, the fluorescence of the samples was analyzed using a plate reader as described below.

The punched corneal triplets were poured into gelan gum (Phytagel, Sigma Aldrich) with 1% concentration and prepared for LSFM measurement. Immediately after a washing step with NaCl solution overnight (4 °C), an ascending alcohol series (30%, 6 h; 50%, overnight; 70%, 6 h; 90%, overnight; 100%, 6 h; 100%, overnight) followed to dehydrate the samples. Incubation in benzyl alcohol / benzyl benzoate (BABB, 1:3 ratio) was performed at room temperature until sufficient tissue transparency was achieved. This process took up to 14 days and led to a refractive index matching of sample and immersion solution to prevent light scattering and light absorption in the immersion chamber of the microscope. After analysis by LSFM, the samples remained in BABB until histological validation.

### Ethics statement

All animal experimental procedures were performed in compliance with the European and German regulations on Animal Welfare and were approved by the administration of Lower Saxony (LAVES) and the Animal Welfare Committee of the University Medical Center Göttingen {No: 20.3554}.

### Sample analysis and data processing

#### Plate reader

As the corneal thickness in all tissue samples were assumed to be comparable, the overall counts of the fluorescence were collected with a plate reader (TriStar2S, Berthold Technologies, Bad Wildbad, Germany; URL: https://www.berthold.com/de/bioanalytik/produkte/mikroplatten-reader/multimode/tristar3/) for analysis of the changes in tissue fluorescence.

For this purpose, the tissue samples that underwent treatment were excited using a xenon lamp and an excitation bandpass filter with a wavelength of λ_ex_ = 485 nm and a bandwidth of 14 nm full width at half maximum (FWHM). Emission was recorded during a detection time of 0.1 s through an emission filter with a center wavelength of λ_em_ = 535 nm and a bandwidth of 25 nm FWHM. The values of the samples with similar treatment (*n* = 12) were used for statistical analysis.

#### Light sheet fluorescence microscopy

The completely cleared corneal samples were analyzed using the UltraMicroscope Blaze™ LSFM (Miltenyi Biotec B.V.& Co KG, Germany). The specimens were attached to the sample holder of the microscope. For the LSFM scanning procedure they were placed into the ethyl cinnamate (ECI) filled cuvette, which was used as a non-corrosive alternative to BABB with comparable refractive index.

Fluorescence excitation was done via NKT SuperK Extreme white light laser of 0.6 W visible power (NKT Photonics A/S, Denmark) and excitation filter. The images were acquired using the software ImSpectorPro 7.5.2. The detection part of the LSFM comprises the objectives (1x, 4x, 12x) and emission filters (Table 2).

**Table 2:** List of all laser band path filter combinations used for LSFM data acquisition.

	Excitation wavelength/bandwidth in nm	Emission wavelength/bandwidth in nm
1	470/40	525/50
2	520/40	585/40

The LSFM is equipped with a 4.2-megapixel sCMOS camera with a 2,048 × 2,048-pixel resolution (PCO AG, Germany). The corresponding filter combinations are listed in Table 2. The first filter combination is in the same range as the filter combination used for the plate reader measurement. The software Zeiss arivis Vision 4D 3.0.1 Pro (Carl Zeiss Microscopy Software, Germany; URL: https://kb.arivis.com/vision4d-arivis-pro) was applied for LSFM data analysis.

#### Validating histology and fluorescence image acquisition

After all, LSFM examinations were completed, corneal tissue samples were placed in xylene for 1.5 h, embedded in paraffin and cut into 2 μm sections for histologic validation. For further staining the tissue slices were deparaffinized (60 °C, 30 min) and rehydrated by a descending ethanol series. The H&E stain and Sirius red stain^39^ were performed according to the manufacturer’s protocol.

Stained tissue slides were imaged by a transmitted light microscope (Zeiss Axioscope II, Carl Zeiss Microscopy GmbH, Germany). For Sirius red detection, the microscope was additionally equipped with polarizing filters (AHF AG, Germany).

Additionally, unstained sections were imaged with a confocal fluorescence microscope (Zeiss LSM 700, Carl Zeiss Microscopy GmbH, Germany). An excitation laser at λ_ex_ = 488 nm and a high pass filter for emission with a cutting wavelength of λ_hp_ = 495 nm was utilized to maintain consistency with the filter combination employed in the plate reader measurement and LSFM acquisition.

#### Statistics

Statistics and statistic-related diagrams were done with SPSS^®^ Statistics (URL: https://www.ibm.com/de-de/products/spss-statistics). Probability values were derived from a pairwise comparison, testing the null hypothesis that the distributions are identical in all sample groups.

## Data Availability

The data used and/or analyzed for the present study as well as the analytical methods can be made available to other researchers on reasonable request to the corresponding author.
